# High quality draft genome sequences of *Pseudomonas fulva* DSM 17717^T^, *Pseudomonas parafulva* DSM 17004^T^ and *Pseudomonas cremoricolorata* DSM 17059^T^ type strains

**DOI:** 10.1186/s40793-016-0178-2

**Published:** 2016-09-01

**Authors:** Arantxa Peña, Antonio Busquets, Margarita Gomila, Magdalena Mulet, Rosa M. Gomila, T. B. K. Reddy, Marcel Huntemann, Amrita Pati, Natalia Ivanova, Victor Markowitz, Elena García-Valdés, Markus Göker, Tanja Woyke, Hans-Peter Klenk, Nikos Kyrpides, Jorge Lalucat

**Affiliations:** 1 Department of Biology-Microbiology, Universitat de les Illes Balears, Campus UIB, Crtra. Valldemossa km 7.5, 07122 Palma de Mallorca, Spain; 2 Serveis Cientifico-Tècnics, Universitat de les Illes Balears, Palma de Mallorca, Spain; 3 DOE Joint Genome Institute, 2800 Mitchell Drive, Walnut Creek, CA 94598-1698 USA; 4 Institut Mediterrani d’Estudis Avançats (IMEDEA, CSIC-UIB), Palma de Mallorca, Spain; 5 Leibniz Institute DSMZ - German Collection of Microorganisms and Cell Cultures, 38124 Braunschweig, Germany; 6 School of Biology, Newcastle University, Newcastle upon Tyne, NE1 7RU UK; 7 Department of Biological Sciences, Faculty of Science, King Abdulaziz University, Jeddah, Saudi Arabia

**Keywords:** Genomic Encyclopedia of Type Strains (GEBA), One Thousand Microbial Genomes Project (KMG-I), *P. fulva*, *P. parafulva*, *P. cremoricolorata*, Genome, Type strains

## Abstract

*Pseudomonas* has the highest number of species out of any genus of Gram-negative bacteria and is phylogenetically divided into several groups. The *Pseudomonas putida* phylogenetic branch includes at least 13 species of environmental and industrial interest, plant-associated bacteria, insect pathogens, and even some members that have been found in clinical specimens. In the context of the *Genomic Encyclopedia of Bacteria and Archaea* project, we present the permanent, high-quality draft genomes of the type strains of 3 taxonomically and ecologically closely related species in the *Pseudomonas putida* phylogenetic branch: *Pseudomonas fulva* DSM 17717^T^, *Pseudomonas parafulva* DSM 17004^T^ and *Pseudomonas cremoricolorata* DSM 17059^T^. All three genomes are comparable in size (4.6**–**4.9 Mb), with 4,119**–**4,459 protein-coding genes. Average nucleotide identity based on BLAST comparisons and digital genome-to-genome distance calculations are in good agreement with experimental DNA-DNA hybridization results. The genome sequences presented here will be very helpful in elucidating the taxonomy, phylogeny and evolution of the *Pseudomonas putida* species complex.

**Electronic supplementary material:**

The online version of this article (doi:10.1186/s40793-016-0178-2) contains supplementary material, which is available to authorized users.

## Introduction

During a taxonomic study of *Pseudomonas* strains isolated from rice, petroleum fields and oil-brine in Japan, Iizuka and Komagata [1] proposed two new species in 1963, *Pseudomonas fulva* and *Pseudomonas straminea* (as cited in Uchino et al. [2]). These new species produced a water-insoluble yellow pigment, but not a water-soluble fluorescent pigment. Later, seven *P. fulva* strains obtained from culture collections were re-characterized and compared with the strains of related species by Uchino and collaborators [2]. Phylogenetic analysis based on 16S rRNA sequences, experimental DNA-DNA hybridization results and phenotypic characteristics led to the proposal of two new species: *Pseudomonas parafulva* (2 strains) and *Pseudomonas cremoricolorata* (1 strain). Three of the four remaining strains were maintained in the species *P. fulva**,* and the other strain was identified as *P. straminaea* [2]. In a multilocus sequence analysis, the type strains of *P. fulva*, *P. parafulva* and *P. cremoricolorata* clustered in the *Pseudomonas putida* phylogenetic branch and are considered members of the *P. putida* group in the *Pseudomonas fluorescens* lineage [3]. The three species are taxonomically and ecologically closely related. Strains from these species have been isolated from rice paddy samples or from Japanese unhulled rice. *P. fulva* strains have also been studied for their endophytic growth in Scots pines and for their roles in plant growth promotion and protection against plant pathogenic fungi [4, 5]. The antagonistic effect against plant pathogenic bacteria has also been demonstrated in other strains of *Pseudomonas putida* [6]. Additionally, *P. fulva* strains have been isolated from water collected from human-made container habitats of mosquitoes [7]. *P. fulva* was one of the most abundant species found in a survey of pseudomonads in human homes [8], and very recently *P. fulva* was identified as a member of a polymicrobial ventriculitis in humans [9]. The difficulty in identifying species closely related to *P. putida* in the clinical laboratory is highlighted by Rebolledo and collaborators [9]. Biosynthesis of medium-chain-length poly(3-hydroxyalkanoates) by a volatile aromatic hydrocarbons-degrading *P. fulva* has been proposed as candidate for the biotechnological conversion of toxic petrochemical wastes to valuable biopolymers [10].

In the context of the *Genomic Encyclopedia of**Bacteria**and Archaea* (GEBA) project [11], the permanent, high quality draft genomes of the type strains of *P. fulva*, *P. parafulva* and *P. cremoricolorata* are presented. The genome sequences have been annotated, and the results are discussed in relation to the taxonomy of members of the *P. putida* phylogenetic group.

## Organisms information

### Classification and features

The type strains of the three species, *P. fulva*DSM 17717^T^ (=JCM 11242^T^ =NRIC 0180^T^), *P. parafulva*DSM 17004^T^ (=AJ 2129^T^ =JCM 11244^T^ =NRIC 0501^T^) and *P. cremoricolorata*DSM 17059^T^ (=JCM 11246^T^ =NRIC 0181^T^), were obtained from the DSMZ. All strains were isolated by Iizuka and Komagata [1, 2] from Japanese rice paddies and were initially proposed as members of the new species *P. fulva* due to the deep yellow color of their colonies. *P. fulva* was included in the Approved Lists of Bacterial Names [12]. Uchino and collaborators re-characterized several strains obtained as *P. fulva* from culture collections and proposed two new species: *P. parafulva* (2 strains) and *P. cremoricolorata* (1 strain) [2].

All three type strains shared the basic phenotypic traits of the genus *Pseudomonas*: Gram-negative rods, motility via polar flagella, with strictly respiratory type of metabolism, catalase and oxidase activity and phylogenetic placement in the genus *Pseudomonas* on the basis of 16S rRNA gene sequencing. None of the three species produced water-soluble fluorescent pigments but produced a characteristic water-insoluble yellow pigment. Colonies appear smooth, round, flat to convex and pale/creamy yellow on nutrient agar. The three species were differentiated from each other by several phenotypic tests: presence of the arginine dihydrolase pathway, growth at 37 °C and assimilation of D-ribose, D-mannose, adonitol, 2-keto-D-gluconate, butyrate, valerate, caprate, isovalerate, itaconate, citraconate, glycerate, levulinate, Tween 80, p-hydroxybenzoate, inosine, glycine, L-ornithine, L-citrulline and nicotinate. An extensive list of phenotypic characteristics can be found in the original publication by Uchino et al. [2]. The classification and general features of *P. fulva*, *P. parafulva* and *P. cremoricolorata* type strains are given in Tables 1, 2 and 3.

**Table 1:** Classification and general features of *P. fulva* DSM 17717^T^ [36]

MIGS ID	Property	Term	Evidence code
	Classification	Domain *Bacteria*	TAS [37]
		Phylum *Proteobacteria*	TAS [38]
		Class *Gammaproteobacteria*	TAS [39]
		Order *Pseudomonadales*	TAS [40]
		Family *Pseudomonadaceae*	TAS [41]
		Genus *Pseudomonas*	TAS [42]
		Species *Pseudomonas fulva*	TAS [2]
		(Type) strain: DSM 17717^T^	
	Gram stain	negative	TAS [2]
	Cell shape	rod-shaped	TAS [2]
	Motility	motile	TAS [2]
	Sporulation	non-sporulating	TAS [2]
	Temperature range	4–37 °C	TAS [2]
	Optimum temperature	30 °C	TAS [2]
	pH range; Optimum	-	NAS
	Carbon source	monosaccharides, organic acids, alcohols, amino acids, amines	TAS [2]
MIGS-6	Habitat	rice paddies	TAS [2]
MIGS-6.3	Salinity	-	NAS
MIGS-22	Oxygen requirement	aerobic	TAS [2]
MIGS-15	Biotic relationship	free-living	TAS [2]
MIGS-14	Pathogenicity	non-pathogen	TAS [2]
MIGS-4	Geographic location	Japan	TAS [2]
MIGS-5	Sample collection	-	NAS
MIGS-4.1	Latitude	-	NAS
MIGS-4.2	Longitude	-	NAS
MIGS-4.4	Altitude	-	NAS

**Table 2:** Classification and general features of *P. parafulva* DSM 17004^T^ [36]

MIGS ID	Property	Term	Evidence code
	Classification	Domain *Bacteria*	TAS [37]
		Phylum *Proteobacteria*	TAS [38]
		Class *Gammaproteobacteria*	TAS [39]
		Order *Pseudomonadales*	TAS [40]
		Family *Pseudomonadaceae*	TAS [41]
		Genus *Pseudomonas*	TAS [42]
		Species *Pseudomonas parafulva*	TAS [2]
		(Type) strain: DSM 17004^T^	
	Gram stain	negative	TAS [2]
	Cell shape	rod-shaped	TAS [2]
	Motility	motile	TAS [2]
	Sporulation	non- sporulating	TAS [2]
	Temperature range	4–37 °C	TAS [2]
	Optimum temperature	30 °C	TAS [2]
	pH range; Optimum	-	TAS [2]
	Carbon source	monosaccharides, organic acids, alcohols, amino acids, amines	TAS [2]
MIGS-6	Habitat	rice paddies	TAS [2]
MIGS-6.3	Salinity	-	NAS
MIGS-22	Oxygen requirement	aerobic	TAS [2]
MIGS-15	Biotic relationship	free-living	TAS [2]
MIGS-14	Pathogenicity	non-pathogen	TAS [2]
MIGS-4	Geographic location	Japan	TAS [2]
MIGS-5	Sample collection	-	NAS
MIGS-4.1	Latitude	-	NAS
MIGS-4.2	Longitude	-	NAS
MIGS-4.4	Altitude	-	NAS

**Table 3:** Classification and general features of *P. cremoricolorata* DSM 17059^T^ [36]

MIGS ID	Property	Term	Evidence code
	Classification	Domain *Bacteria*	TAS [37]
		Phylum *Proteobacteria*	TAS [38]
		Class *Gammaproteobacteria*	TAS [39]
		Order *Pseudomonadales*	TAS [40]
		Family *Pseudomonadaceae*	TAS [41]
		Genus *Pseudomonas*	TAS [42]
		Species *Pseudomonas cremoricolorata*	TAS [2]
		(Type) strain: DSM 17059^T^	
	Gram stain	negative	TAS [2]
	Cell shape	rod-shaped	TAS [2]
	Motility	motile	TAS [2]
	Sporulation	non-sporulating	TAS [2]
	Temperature range	4–30 °C	TAS [2]
	Optimum temperature	30 °C	TAS [2]
	pH range; Optimum	-	NAS
	Carbon source	monosaccharides, organic acids, alcohols, amino acids, amines	TAS [2]
MIGS-6	Habitat	rice paddies	TAS [2]
MIGS-6.3	Salinity	-	NAS
MIGS-22	Oxygen requirement	aerobic	TAS [2]
MIGS-15	Biotic relationship	free-living	TAS [2]
MIGS-14	Pathogenicity	non-pathogen	TAS [2]
MIGS-4	Geographic location	Japan	TAS [2]
MIGS-5	Sample collection	-	NAS
MIGS-4.1	Latitude	-	NAS
MIGS-4.2	Longitude	-	NAS
MIGS-4.4	Altitude	-	NAS

#### Chemotaxonomic data

As reported by Uchino and collaborators [2] the DNA GC-content of the three type strains, as determined by chemical analysis, was 60.0 mol % in *P. fulva* and *P. parafulva* and 62.1 mol % in *P. cremoricolorata*. The percentages of G + C bases based on the genome analysis were 61.71 % for *P. fulva*DSM 17717^T^, 62.42 % for *P. parafulva*DSM 17004^T^ and 63.47 % for *P. cremoricolorata*DSM 17059^T^. The GC-contents determined by chemical analysis were slightly lower than those inferred from genome sequences. The predominant respiratory quinone was ubiquinone Q-9, but Q-8 and Q-10 were also detected in smaller amounts. The major cellular fatty acids were C16:0, C16:1 and C18:1, and the major 3-hydroxy fatty acids were C10:0 and C12:0 [2].

For protein analysis, cells were cultured in Luria-Bertani broth aerobically, with shaking at 30 °C, harvested in the exponential growth phase and prepared for Whole-cell MALDI-TOF MS analysis using an Autoflex III mass spectrometer (Bruker Daltonik, Germany) as recommended by the manufacturer. Protein profiles clearly distinguished the type strains in the *P. putida* phylogenetic group [3]. A list of major proteins that met a minimum intensity threshold of 700, a minimum signal to noise threshold of 15, and a mass to charge ratio (m/z) higher than 3,000 and lower than 10,000 is included in Additional file 1.

### Extended feature descriptions

**Fig. 1:**
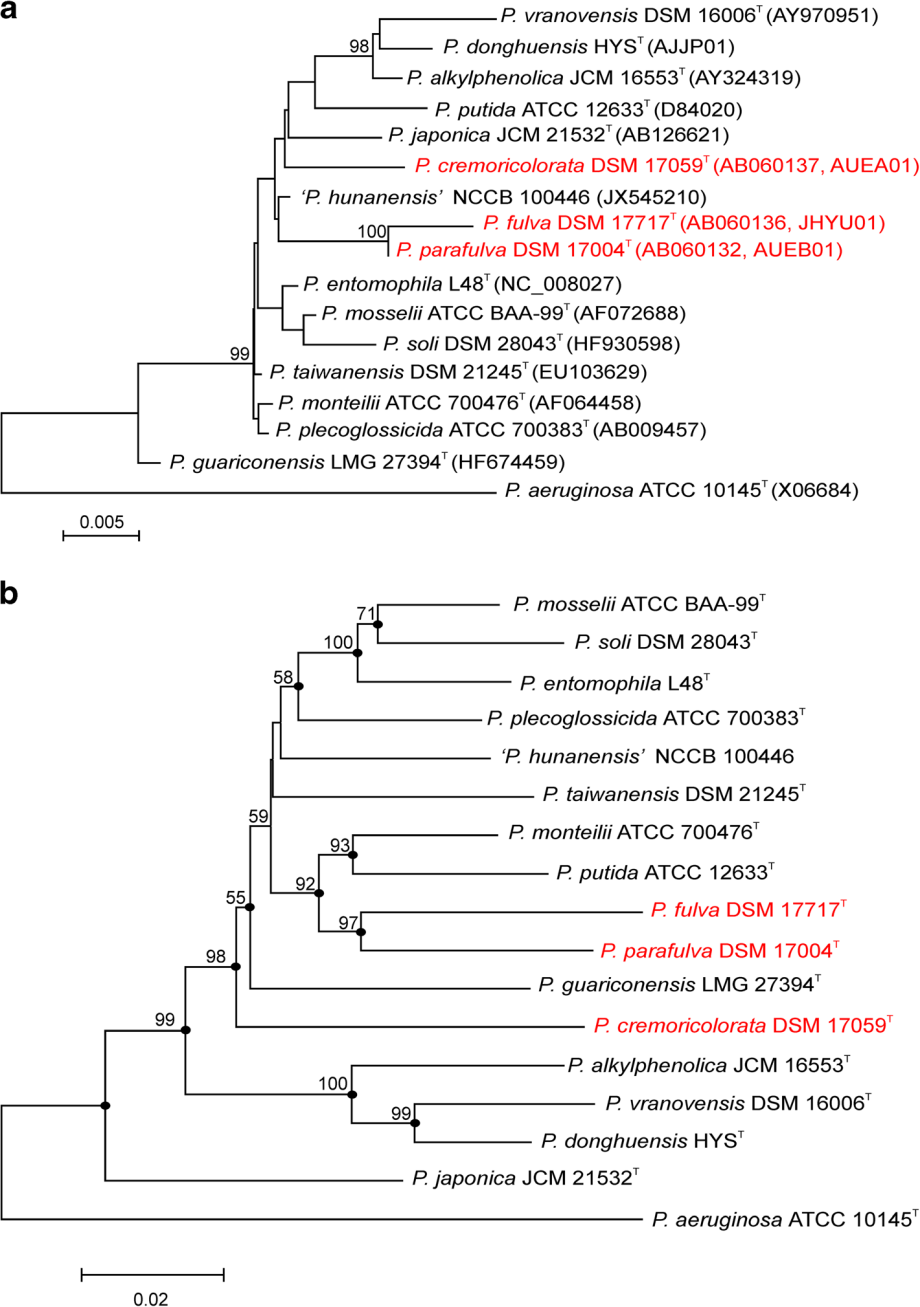
**a** Phylogenetic tree based on the almost complete sequence of the 16S rRNA gene of the type strains of the species in the *P. putida* phylogenetic group. Evolutionary distances were computed with MEGA (version 6) using the Jukes-Cantor method [13]. Dendrogram was generated by the Neighbor-Joining method. The bar indicates the number of base substitutions per site (1264 positions in final dataset). Percentage bootstrap values above 50 % (from 1000 replicates) are indicated at the nodes. **b** Phylogenetic tree based on concatenated 16S rRNA, *gyrB* and *rpoD* partial gene sequences of the type strains of species in the *P. putida* phylogenetic group. Evolutionary distances were computed with MEGA (version 6) using the Jukes-Cantor method [13]. Dendrogram was generated by the Neighbor-Joining method. The bar indicates the number of base substitutions per site (2758 positions in the final dataset). Percentage bootstrap values above 50 % (from 1000 replicates) are indicated at the nodes. The black dots indicate nodes maintained in all phylogenetic trees tested

Phylogenetic trees were reconstructed using different methods, namely the maximum-likelihood, maximum-parsimony and neighbor-joining algorithms integrated in MEGA version 6 bioinformatics package [13], and also using the FastME 2.0 phylogeny inference program [14]. All phylogenetic trees tested showed similar topologies and the same strain groupings. The derived phylogeny of the species in the *P. putida* phylogenetic group based on 16S rDNA gene sequencing had low resolution, and the bootstrap values of branches were low (Fig. 1a). Therefore, a phylogenetic tree based on a multilocus sequence analysis with the partial sequences of three housekeeping genes (16S rDNA, *gyrB*, and *rpoD*) was constructed as recommended by Mulet et al. [3] (Fig. 1b). Most branches were supported in the MLSA phylogenetic tree by high bootstrap values, and all type strains were clearly separated in the *P. putida* phylogenetic group. The strain groupings (*P. putida*/*Pseudomonas monteilii*/*P. parafulva*/*P. fulva*; *Pseudomonas soli*/*Pseudomonas mosselii*/*Pseudomonas entomophila*/*Pseudomonas plecoglossicida* and *Pseudomonas donghuensis*/*Pseudomonas vranovensis*/*Pseudomonas alkylphenolica*) were maintained in all trees. ‘*Pseudomonas hunanensis**’*NCCB 100446 (proposed as a new species [15], but not yet validated) and *Pseudomonas taiwanensis*DSM 21245^T^ branches were supported by low bootstrap values, and their positions varied in the trees.

## Genome sequencing information

### Genome project history

Sequencing of the three type strains is part of Genomic Encyclopedia of Type Strains, Phase I: the one thousand microbial genomes (KMG-I) project [16], a follow-up of the GEBA pilot project [11, 17]. Project information is deposited in the Genomes on Line Database (GOLD) [18], and the high quality draft genome sequence is deposited in GenBank and in the Integrated Microbial Genomes database (IMG) [19]. Draft sequencing, initial gap closure and annotation were performed by the DOE Joint Genome Institute (JGI) using state-of-the-art sequencing technology [20]. A summary of the project information is shown in Table 4. Genbank IDs are as follows: JHYU00000000 for *P. fulva*DSM 17717^T^, AUEB00000000 for *P. parafulva*DSM 17004^T^ and AUEA00000000 for *P. cremoricolorata*DSM 17059^T^.

**Table 4:** Project information for *P. fulva* DSM 17717^T^, *P. parafulva* DSM 17004^T^, and *P. cremoricolorata* DSM 17059^T^

		*P. fulva* DSM 17717^T^	*P. parafulva* DSM 17004^T^	*P. cremoricolorata* DSM 17059^T^
MIGS ID	Property	Term	Term	Term
MIGS 31	Finishing quality	Permanent Draft, High-quality draft	Permanent Draft, High-quality draft	Permanent Draft, High-quality draft
MIGS-28	Libraries used	Illumina Regular Fragment, 270bp	Illumina Regular Fragment, 270bp	Illumina Regular Fragment, 270bp
MIGS 29	Sequencing platforms	Illumina HiSeq 2000, Illumina HiSeq 2500	Illumina HiSeq 2000, Illumina HiSeq 2500	Illumina HiSeq 2000, Illumina HiSeq 2500
MIGS 31.2	Fold coverage	Unknown	Unknown	Unknown
MIGS 30	Assemblers	vpAllpaths v. r46652	Unknown program v. before 2013–03–26	Unknown program v. before 2013–03–26
MIGS 32	Gene calling method	Prodigal 2.5	Prodigal 2.5	Prodigal 2.5
	Locus Tag	Q382	H619	H618
	Genbank ID	JHYU00000000	AUEB00000000	AUEA00000000
	GenBank Date of Release	May 5, 2014	Dec 12, 2014	Dec12, 2013
	GOLD ID	Gi0045700	Gp0021954	Gi18319
	BIOPROJECT	PRJNA221053	PRJNA188912	PRJNA188911
	IMG Taxon ID	2556921649	2523533547	2523533534
MIGS 13	Source Material Identifier	DSM 17717	DSM 17004	DSM 17059
	Project relevance	GEBA-KMG, Tree of Life	GEBA-KMG, Tree of Life	GEBA-KMG, Tree of Life

### Growth conditions and genomic DNA preparation

*P. fulva*DSM 17717^T^, *P. parafulva*DSM 17004^T^ and *P. cremoricolorata*DSM 17059^T^ were cultured aerobically in Luria-Bertani medium, with shaking at 30°C, to the early stationary phase. Genomic DNA was extracted and purified with a Promega Wizard® Genomic DNA Purification kit, following the manufacturer’s instructions. DNA quality and quantity were determined with a Nanodrop spectrometer (Thermo Scientific, Wilmington, USA).

### Genome sequencing and assembly

An Illumina standard shotgun library was constructed and sequenced using the Illumina HiSeq 2000 platform. For *P. fulva*, 16,075,374 reads were generated, totaling 2,411.3 Mb, of which 1,500 Mb were used in an assembly, resulting in an average coverage of 312.5x. For *P. parafulva*, 14,015,748 reads were generated, totaling 2,102.4 Mb, of which 607.0 Mb were used in an assembly, resulting in an average coverage of 122x. For *P. cremoricolorata*, 11,621,460 reads were generated, totaling 1,743.2 Mb, of which 569.4 Mb were used in an assembly, resulting in an average coverage of 122x. Illumina sequencing and library artifacts were removed using Duk filtering (L. Mingkun, A. Copeland, and H. J. Duk, unpublished data). Filtered Illumina reads were assembled using Velvet (version 1.1.04) [21], simulated paired-end reads were created from Velvet contigs using wgsim and simulated read pairs were reassembled using Allpaths-LG (version r42328) [22].

### Genome annotation

Protein-coding genes were identified using Prodigal [23], as part of the DOE-JGI genome annotation pipeline [24]. Additional gene prediction analysis and manual functional annotation were performed within the Integrated Microbial Genomes (IMG) platform, which provides tools for analyzing and reviewing the structural and functional annotations of genomes in a comparative context [19]. Genome annotation procedures are detailed in Markowitz et al. [19] and references therein. Briefly, the predicted CDSs were translated and used to search the NCBI nonredundant database, UNIProt, TIGRFam, Pfam, KEGG, COG and InterPro databases. Transfer RNA genes were identified using the tRNAScan-SE tool and other non-coding RNAs were found using INFERNAL. Ribosomal RNA genes were predicted using hmmsearch against the custom models generated for each type of rRNA.

## Genome properties

The assembly of the three genomes consisted of 4.7 Mb in 48 scaffolds for *P. fulva*, 4.9 Mb in 33 scaffolds for *P. parafulva* and 4.6 Mb in 27 scaffolds for *P. cremoricolorata* (Table 5). The G + C content for each strain was 61.72, 62.42 and 63.47 %, respectively. The majority of protein-coding genes (78.96, 80.59 and 79.68 %) were assigned a putative function. The properties and statistics of the genomes are summarized in Table 5, and the number of genes associated with general COG functional categories is shown in Table 6.

**Table 5:** Genome statistics for *P. fulva* DSM 17717^T^, *P. parafulva* DSM 17004^T^ and *P. cremoricolorata* DSM 17059^T^

Attribute	*P. fulva* DSM 17717^T^		*P. parafulva* DSM 17004^T^		*P. cremoricolorata* DSM 17059^T^	
	Value	% of Total^a^	Value	% of Total^a^	Value	% of Total^a^
Genome size (bp)	4,770,636	100.00	4,958,587	100.00	4,660,374	100.00
DNA coding (bp)	4,280,442	89.72	4,475,423	90.26	4,196,318	90.04
DNA G + C (bp)	2,943,912	61.72	3,095,099	62.42	2,958,082	63.47
DNA scaffolds/contigs^b^	48/54	100.00	33/40	100.00	27/27	100.00
Total genes	4,397	100.00	4,575	100.00	4,238	100.00
Protein-coding genes	4,278	97.29	4,459	97.46	4,119	97.19
RNA genes	119	2.71	116	2.54	119	2.81
Pseudo genes	0	0.00	0	0.00	0	0.00
Genes in internal clusters	448	10.19	421	9.20	381	8.99
Genes with function prediction	3,472	78.96	3,687	80.59	3,377	79.68
Genes assigned to COGs	3,162	71.91	3,371	73.68	3,074	72.53
Genes with Pfam domains	3,707	84.31	3,892	85.07	3,588	84.66
Genes with signal peptides	443	10.08	476	10.40	448	10.57
Genes with transmembrane helices	941	21.40	992	21.68	910	21.47
CRISPR repeats	0	0.00	0	0.00	0	0.00

^a^The total is based on either the size of the genome in base pairs or the total number of protein coding genes in the annotated genome

^b^Number of DNA scaffolds and contigs, available in the JGI website and NCBI databases, respectively

**Table 6:** Number of genes associated with general COG functional categories for *P. fulva* DSM 17717^T^, *P. parafulva* DSM 17004^T^, and *P. cremoricolorata* DSM 17059^T^

Code	*P. fulva* DSM 17717^T^		*P. parafulva* DSM 17004^T^		*P. cremoricolorata* DSM 17059^T^		Description
	Value	% age	Value	% age	Value	% age	
J	225	5.12	237	5.18	230	5.43	Translation, ribosomal structure and biogenesis
A	1	0.02	1	0.02	1	0.02	RNA processing and modification
K	281	6.39	317	6.93	274	6.47	Transcription
L	115	2.62	121	2.64	116	2.74	Replication, recombination and repair
B	2	0.05	4	0.09	1	0.02	Chromatin structure and dynamics
D	37	0.84	38	0.83	38	0.90	Cell cycle control, Cell division, chromosome partitioning
V	70	1.59	78	1.70	69	1.63	Defense mechanisms
X	32	0.73	30	0.66	44	1.04	Mobilome: prophages, transposons
W	17	0.39	17	0.37	13	0.31	Extracellular structures
T	246	5.59	267	5.84	230	5.43	Signal transduction mechanisms
M	202	4.59	220	4.81	208	4.91	Cell wall/membrane biogenesis
N	107	2.43	110	2.40	97	2.29	Cell motility
U	61	1.39	56	1.22	73	1.72	Intracellular trafficking and secretion
O	137	3.12	140	3.06	137	3.23	Posttranslational modification, protein turnover, chaperones
C	240	5.46	253	5.53	212	5.00	Energy production and conversion
G	170	3.87	173	3.78	151	3.56	Carbohydrate transport and metabolism
E	390	8.87	427	9.33	366	8.64	Amino acid transport and metabolism
F	88	2.00	94	2.05	78	1.84	Nucleotide transport and metabolism
H	203	4.62	209	4.57	195	4.60	Coenzyme transport and metabolism
I	168	3.82	174	3.80	169	3.99	Lipid transport and metabolism
P	213	4.84	240	5.25	232	5.47	Inorganic ion transport and metabolism
Q	79	1.80	96	2.10	89	2.10	Secondary metabolites biosynthesis, transport and catabolism
R	297	6.75	311	6.80	280	6.61	General function prediction only
S	192	4.37	198	4.33	181	4.27	Function unknown
-	1235	28.09	1204	26.32	1164	27.47	Not in COGs

The total is based on the total number of protein coding genes in the genome

## Insights from the genome sequence

Experimental DNA-DNA hybridizations were performed by Uchino et al. [2], following the fluorometric procedure proposed by Ezaki et al. [25]. Taxonomic genome comparisons were calculated by two different procedures: Average nucleotide identity based on BLAST was calculated with the JSpecies program [26]. Digital DDH similarities among the genomes of the three type strains were calculated using GGDC web server version 2.0 [27] under recommended settings. The results are given in Table 7 and are highly consistent. Experimental and dDDH values were clearly below the 70 species threshold, and ANIb was below the accepted 95–96 % species threshold. The type strains *P. fulva*NBRC 16637^T^ and *P. parafulva*NBRC 16636^T^ have been sequenced at the NBRC, and the ANIb values between the genomes of the type strain pair *P. fulva*DSM 17717 ^T^ and *P. fulva*NBRC 16637^T^ were in 99.98 % agreement; the dDDH values were 100 % identical. The same results were obtained when comparing the type strain pair *P. parafulva*DSM 17004^T^ and *P. parafulva*NBRC 16636^T^ (99.95 and 100 % similarity for ANIb and GGDC results, respectively). Using the whole-genome ANI-based MiSI method [28], which is computed for all bacterial genomes in the Integrated Microbial Genomes system, *P. fulva*DSM 17717^T^ clustered in the same gANI clique with eight plant-associated genome-sequenced *Pseudomonas* sp. not yet classified at the species level, with an intra-clique ANI of 99.57 %, indicating that the 9 strains belong genomically to the same species, *P. fulva*. The strain *P. fulva*NBRC 16637^T^ is the equivalent type strain of the NITE (Biological Resource Center) and was included in the same clique. The GC-content variation within the clique was less than 1 % (61.58 %–61.88 %), which is proof of the value of draft genomes for taxonomy because the GC-content varies no more than 1 % within species [29], and all strains in the clique should be considered strains in the same genomic species [28]. Three additional genomes of strains identified as *P. fulva*, *P. parafulva* and *P. cremoricolorata*, available in the Genbank database on June 17, 2015, were also analyzed. The completely sequenced genome of *P. cremoricolorata* ND07 (CP009455) showed ANIb and dDDH values of 92 and 50 %, respectively, with *P. cremoricolorata*DSM 17059^T^, indicating a close relationship that is below the species threshold. The complete genome of *P. parafulva* CRS01-1 (CP009747) showed an ANIb value of 81 % with the type strain of *P. parafulva*, the closest related type strain. Finally, as was previously documented, the complete sequenced genome of strain *P. fulva* 12-X (CP002727) demonstrated that it is clearly a distinct species, with an ANIb value of 75.24 % [30] with the *P. fulva* type strain. In all three cases, the genome comparisons did not support a correct species affiliation of the strains.

**Table 7:** Experimental and digital genome similarities calculated for the *P. fulva* DSM 17717^T^, *P. parafulva* DSM 17007^T^ and *P. cremoricolorata* DSM 17059^T^ type strains

	*P. fulva* DSM 17717^T^			*P. parafulva* DSM 17007^T^			*P. cremoricolorata* DSM 17059^T^		
	exp^a^	ANIb	dDDH	exp	ANIb	dDDH	exp	ANIb	dDDH
*P. fulva* DSM 17717 ^T^	100	100	100	34	82.44	26.20	24	79.49	23.70
*P. parafulva* DSM 17007^T^	43	82.44	26.20	100	100	100	32	80.08	24.30
*P. cremoricolorata* DSM 17059 ^T^	39	79.49	23.70	34	80.08	24.30	100	100	100

Data are given in percentage. Experimental data have been retrieved from Uchino et al. [2]

^a^Experimental results

The presence of genes related to carbohydrate and amino acid transport and metabolism is relevant for the fitness of environmental bacteria. These genes represent 12–13 % of the total genes detected in the three strains, and they also have taxonomic consequences. Substrate utilization is an essential criterion for *Pseudomonas* taxonomy, and several tests routinely used in *Pseudomonas* identifications have been employed in the present study.

Catalase and superoxide dismutase are relevant enzymes for protecting the cell against reactive oxygen and are characteristic of most *Pseudomonas*. Catalase activity was detected by Uchino et al. [2] in *P. fulva*, *P. parafulva*, and *P. cremoricolorata**.* Accordingly, 3 genes potentially coding for catalase were found in *P. fulva* and *P. parafulva*, but only 2 were found in *P. cremoricolorata*; three genes coding for superoxide dismutase were detected in *P. fulva* and *P. cremoricolorta* genomes, but only two were found in *P. parafulva*. Testing for the presence of the arginine dihydrolase (or arginine deiminase) pathway, in combination with other biochemical tests, can also be of diagnostic value [31]. The arginine dihydrolase pathway transforms arginine to ornithine with ATP gain and allows limited growth in several *Pseudomonas* under anaerobic conditions. The *arcA* gene is present in the *P. fulva* and *P. parafulva* genomes but is absent in *P. cremoricolorata*, in accordance with the experimental data obtained by Uchino et al. [2]. All three strains were negative for nitrate reduction, nitrate respiration and PHB synthesis, and, accordingly, no gene related to these pathways was detected in any of the genomes. Cleavage of aromatic compounds was also tested using protocatechuate as a substrate; a gene coding for the protocatechuate 3,4-dioxygenase (3-oxoadipate pathway) was found in *P. fulva* and *P. parafulva* but was absent in *P. cremoricolorata*, confirming the ortho cleavage of the aromatic ring as reported by Uchino and collaborators [2]. All three strains possessed genes encoding key enzymes involved in glucose catabolism via the glycolysis, pentose-phosphate and 2-keto-3-deoxi-6 phosphogluconate pathways. The three species were recorded as amylase negative, but an alpha-amylase gene (*amyA*) was detected in all three genomes, indicating the potential ability to grow with starch as a substrate.

Bacterial secretion systems transport proteins across the cell envelope of Gram-negative bacteria to the external milieu and are considered critical for persistence in an ecological niche and for conquering a new one [32]. Type VI secretion system seems to be the most common and appears to be confined to proteobacteria. The TVISS consists of 13 essential conserved genes, many of which contain a number of functionally accessory elements. Several TVISS are often present in a single genome [33]. They have been mainly studied for their pathogenic role in the interaction between bacteria and hosts, but TVISS seems to play a role in mutualistic relationships between bacteria and eukaryotic cells or between bacteria, as well. A set of 15 conserved TVISS genes were found in *P. fulva*DSM 17717^T^ but were absent in the other two strains. *P. fulva*DSM 17717^T^ also has three copies of a Rhs element Vgr protein not present in the other strains that can be exported by the TVISS, but its exact function is still not known. The possible role of TVISS genes in the pathogenesis or in the interactions of *P. fulva*DSM 17717^T^ with the environment remains to be elucidated.

Prophage-like elements in microbial genomes represent one of the main contributors of mobile DNA, also known as the mobilome [34], and are the main reason for bacterial intraspecies variability. The prophage contribution to the bacterial genome is highly variable. It can represent up to 8 % of the total chromosomal DNA [35], but phages may also be absent. The mobilomes of *P. fulva*, *P. parafulva* and *P. cremoricolorata* were predicted to contain 32, 30, and 44 genes, respectively. In addition to transposases, integrases and regulatory elements, clusters of bacteriophage structural genes (6 to 13 genes in a cluster) were found in the 3 strains: 2 clusters in *P. fulva*DSM 17717^T^ (6 and 12 genes in each cluster), 2 in *P. parafulva*DSM 17004^T^ (12 and 9 genes) and 3 in *P. cremoricolorata*DSM 17059^T^ (9, 13 and 10). CRISPR elements were not detected.

## Conclusions

Genome comparisons confirmed the distinct species status of the three type strains analyzed, as well as the close relationships between them. The complete genome analysis also revealed important taxonomic results, highlighting the relevance of the correct species assignation of strains and the need for the genome sequences of species type strains to build a phylogenomic taxonomy. No discrepancies were found between the genome insights and the phenotypic traits previously published for the species. However, the gene content revealed potential properties not yet detected, such as the presence of secretion systems, whose relevance remains to be explored. The genome sequences of the three type strains will be very helpful in elucidating the phylogeny and evolution of the *P. putida* species complex, a relevant coherent group of closely-related species with important ecological and biotechnological implications.

## Additional file

Additional file 1: Major protein profiles of *P. fulva* DSM 17717^T^, *P. parafulva* DSM 17004^T^ and *P. cremoricolorata* DSM 17059^T^ type strains. The intensity value was determined as an average from all spectra containing that peak and the relative intensities with respect to the base peak in percentage are indicated within parenthesis. (m/z: mass to charge ratio; + and -: presence or absence of the corresponding protein). (PDF 99 kb)

## Abbreviations

ANIb: Average nucleotide identity based on BLAST
dDDH: Digital DNA-DNA hybridization
DDH: DNA-DNA hybridization
gANI: Whole-genome ANI-based MiSI method
GEBA: Genomic encyclopedia of Bacteria and Archaea
KMG: One thousand microbial genomes
MIGS: Minimum information about a genome sequence
MLSA: Multilocus sequence analysis
NAS: Non-traceable
TAS: Traceable.
