# Supplementary material for: High quality draft genome sequences of Pseudomonas fulva DSM 17717T, Pseudomonas parafulva DSM 17004T and Pseudomonas cremoricolorata DSM 17059T type strains
Source: Stand Genomic Sci. 2016 Sep 1;11(1):55. doi: 10.1186/s40793-016-0178-2 (PMC5009691; doi:10.1186/s40793-016-0178-2)
Supplement: Additional file 1: — Major protein profiles of P. fulva DSM 17717T, P. parafulva DSM 17004T and P. cremoricolorata DSM 17059T type strains. The intensity value was determined as an average from all spectra containing that peak and the relative intensities with respect to the base peak in percentage are indicated within parenthesis. (m/z: mass to charge ratio; + and -: presence or absence of the corresponding protein). (PDF 99 kb) [file 40793_2016_178_MOESM1_ESM.pdf]

Additional file 1. Major protein profiles of *P. fulva* DSM 17717^T, *P. parafulva* DSM 17004^T and *P. cremoricolorata* DSM 17059^T type strains. The intensity value was determined as an average from all spectra containing that peak and the relative intensities with respect to the base peak in percentage are indicated within parenthesis. (m/z: mass to charge ratio; + and -: presence or absence of the corresponding protein).

**m/z**	***P. fulva*** **DSM 17717^T**	***P. parafulva*** **DSM 17004^T**	***P. cremoricolorata*** **DSM 17059^T**
3312.986	-	+ (27.07)	+ (3.80)
3320.410	+ (30.10)	-	-
3327.529	-	-	+ (25.95)
3586.468	+ (26.30)	+ (14.35)	+ (21.05)
3608.237	+ (21.72)	+ (19.97)	+ (20.75)
4120.252	+ (19.73)	+ (14.13)	+ (16.92)
4434.465	+ (23.52)	+ (30.12)	-
4449.040	-	-	+ (36.12)
4944.209	+ (18.97)	+ (13.02)	-
4962.221	+ (13.65)	-	-
4969.624	-	-	+ (15.75)
4976.256	-	+ (15.13)	-
5109.038	+ (11.75)	-	+ (14.15)[a]
5115.229	-	+ (11.78)	-
5139.079	+ (99.03)	+ (100)	+ (100)
5643.926	+ (5.72)	-	-
5990.967	+ (45.12)	+ (45.03)	+ (40.65)
6292.116	+ (6.90)	+ (7.27)	-
6639.057	+ (51.83)	-	-
6653.040	-	-	+ (43.78)
7173.123	+ (45.70)	+ (27.52)	+ (37.65)
7214.484	+ (28.40)	+ (26.95)	+ (26.33)
8240.842	+ (13.62)	+ (9.95)	+ (11.52)
9891.023	+ (7.68)	+ (5.20)	-

[a]This protein is present in 4 of the 6 replicates; all the other proteins listed in the table are present in the 6 replicates.
